# Group membership does not modulate automatic imitation

**DOI:** 10.1007/s00426-021-01526-1

**Published:** 2021-06-09

**Authors:** Oliver Genschow, Mareike Westfal, Emiel Cracco, Jan Crusius

**Affiliations:** 1 Social Cognition Center Cologne, University of Cologne, Richard-Strauss Str. 2, 50931 Cologne, Germany; 2 Department of Experimental Psychology, Ghent University, Ghent, Belgium

## Abstract

Individuals have the automatic tendency to imitate each other. A key prediction of different theories explaining automatic imitation is that individuals imitate in-group members more strongly than out-group members. However, the empirical basis for this prediction is rather inconclusive. Only a few experiments have investigated the influence of group membership using classic automatic imitation paradigms and these experiments led to mixed results. To put the group membership prediction to a critical test, we carried out six high-powered experiments (total *N* = 1538) in which we assessed imitation with the imitation-inhibition task and manipulated group membership in different ways. Evidence across all experiments indicates that group membership does not modulate automatic imitation. Moreover, we do not find support for the idea that feelings of affiliation or perceived similarity moderate the effect of group membership on automatic imitation. These results have important implications for theories explaining automatic imitation and contribute to the current discussion of whether automatic imitation can be socially modulated.

**Supplementary Information:**

The online version contains supplementary material available at 10.1007/s00426-021-01526-1.

## Introduction

Individuals have the propensity to automatically imitate a wide range of different behaviors, such as facial expressions (Dimberg, 1982), emotions (Hess & Fischer, 2016), postures (LaFrance, 1982), gestures (Cracco, Genschow, et al., 2018), and simple movements (Genschow & Florack, 2014; Genschow & Schindler, 2016; Genschow et al., 2013; Genschow, Hansen, et al., 2019). The most-often used task to study automatic imitation is the imitation-inhibition task (Brass et al., 2000, 2001; for a meta-analysis see Cracco, Bardi, et al., 2018). In this task, participants are instructed to respond over many trials to two imperative cues with two different finger lifting movements. For instance, participants have to respond to the number “1” or “2” by lifting their index or middle finger. At the same time, participants see on a computer screen another person lifting either the same (i.e., congruent movement) or the other finger (i.e., incongruent movement). The typical finding in such an automatic imitation paradigm is that individuals respond faster and more accurately on congruent trials as compared with incongruent trials. Past research has demonstrated that the imitation-inhibition task is a valid (Cracco & Brass, 2019) and robust (Cracco, Bardi, et al., 2018) measure of imitation and produces larger as well as more reliable effects than other imitation tasks (Genschow et al., 2017).

Classic perception–action theories (e.g., Chartrand & Bargh, 1999; Dijksterhuis & Bargh, 2001; Greenwald, 1970; Prinz, 1990, 1997) explain automatic imitation with the notion that the observation and execution of an action activate similar motor representations. This shared representation then increases the likelihood that observing an action leads to the execution of the very same action. The idea that observing an action activates the corresponding motor plan in the observer has been supported by many different findings, including behavioral studies (e.g., Brass et al., 2000, 2001; Craighero et al., 2002; Kilner et al., 2003), fMRI (e.g., Gazzola & Keysers, 2009; Keysers & Gazzola, 2010), motor TMS (e.g., Catmur et al., 2007; Fadiga et al., 1995), as well as single-cell recordings in monkeys (Di Pellegrino et al., 1992) and humans (Mukamel et al., 2010).

While the above-reviewed literature suggests a direct link between observed and executed actions, other research suggests that this link depends on social contexts (Duffy & Chartrand, 2015; Wang & Hamilton, 2012). One of the most-often discussed social moderators of automatic imitation is group membership. Belonging to a social group and establishing stable and cohesive bonds with members from the in-group has an evolutionary important impact on human life (Baumeister & Leary, 1995; Dunbar, 2012; Dunbar & Shultz, 2010) and recognizing a member of the in-group, such as a person from the same ethnical group for instance, affects perceived distance to this person (Fini et al., 2020) and elicits a motivation to affiliate with this person (Van Der Schalk et al., 2011). In line with this notion, previous research on automatic imitation suggests that members from the in-group are more strongly imitated than members from the out-group (e.g., Genschow & Schindler, 2016; Gleibs et al., 2016). Different theoretical accounts have been put forward to explain this, but also other, social modulations.

Motivational theories explain why group membership influences automatic imitation (Chartrand & Dalton, 2009; Wang & Hamilton, 2012) by arguing that people imitate others to gain social benefits. Support for this idea comes from studies showing that being imitated causes people to feel closer (van Baaren et al., 2004) and more affiliated to the imitator (Lakin & Chartrand, 2003), and to behave in a more prosocial manner (Lakin et al., 2008; van Baaren et al., 2004). Based on these results, motivational theories argue that people use imitation, either consciously or unconsciously, to affiliate with others. Because individuals have the general tendency to affiliate more with the in-group than with the out-group (Van Der Schalk et al., 2011), people imitate in-group members more strongly than out-group members.

Although motivational theories can explain why social group membership modulates imitation, they do not explain how this modulation is implemented. A framework that addresses the how-question is the dual-route framework (Heyes, 2011). This account argues that automatic imitation can be (socially) modulated either by input or output modulation. Input modulation refers to the degree to which action observation activates corresponding motor representations. An important factor influencing how strongly observed actions activate the motor system is attention (e.g., Chong et al., 2009). Hence, individuals imitate out-group members less, because they pay less attention to the actions of out-group members than to the actions of in-group members. Another factor that operates at the input level is similarity. Motor learning theories (e.g., Brass & Heyes, 2005; Greenwald, 1970; Heyes, 2010; Prinz, 1990, 1997) argue that imitative tendencies are learned responses that develop as a result of self-observation and interaction with other, often similar (Efferson et al., 2008), individuals (Brass & Heyes, 2005; Cook et al., 2014; Heyes, 2010; Ray & Heyes, 2011). Thus, in-group members are imitated more than out-group members, because in-group members are perceived as more similar than out-group members both at a physical (Press, 2011) and a conceptual level (Cracco, Bardi, et al., 2018). Output modulation, on the other hand, refers to how strongly motor activation elicited by action observation exerts an influence on behavior. Such an account would suggest that actions performed by in- and out-group members both activate the motor system similarly, but that imitative responses elicited by out-group members are subsequently inhibited.

Interestingly, despite the different explanations for the influence of group membership on automatic imitation, the empirical evidence for the group membership effect is rather unclear.

## Empirical evidence for the link between group membership and automatic imitation

On the one hand, some researchers found that individuals imitate in-group members more strongly than out-group members (Genschow & Schindler, 2016; Gleibs et al., 2016). However, this effect was only found when participants felt affiliated with the in-group (Genschow & Schindler, 2016) or when they were in a cooperative as compared with a competitive mindset (Gleibs et al., 2016). On the other hand, some researchers found in one experiment the exact opposite; meaning that participants imitated out-group members stronger than in-group members (Rauchbauer et al., 2015). However, in another experiment, the same authors found this effect only when participants were imitating target persons who displayed angry facial expression (Rauchbauer et al., 2016). Finally, recent research did not find any difference between the imitation of in- and out-group members in a multiple agent paradigm (De Souter et al., 2021; for similar results, see Weller et al., 2020).

Taken together, previous research produced rather mixed results with respect to the question of whether automatic imitation is modulated by group membership or not. Strikingly, each of the conducted experiments has limitations that hinder a clear conclusion of whether group membership modulates automatic imitation. That is, several experiments manipulated other factors such as emotions (Rauchbauer et al., 2015, 2016) or a cooperation vs. competition mindset (Gleibs et al., 2016) on top of group membership leaving open whether imitation itself is influenced by group membership. Other experiments (e.g., Genschow & Schindler, 2016) assessed only a small number of participants leaving open whether the basic effect is replicable. Finally, some of the experiments (De Souter, 2021; Gleibs et al., 2016) manipulated group membership with minimal group paradigms that are known to produce smaller effects than natural groups, such as ethnic groups for example (Ostrom & Sedikides, 1992). Thus, to set the group membership prediction to a stronger test, in the present research, we assessed within six high-powered experiments the classic imitation-inhibition task (Brass et al., 2000, 2001) by presenting hands from in- and out-group members belonging to different nationalities (Experiments 1–4) or ethnic groups (Experiments 5 and 6).

## Present research

The goal of the present research was to test the hypothesis that automatic imitation is stronger for in-group members than for out-group members. In addition, we assessed two different moderating variables to shed light onto potential processes underlying the influence of group membership on automatic imitation and to test different theoretical accounts that had been put forward to explain social modulations of imitation. First, we tested whether feeling affiliated with the in-group moderates automatic imitation. Motivational theories argue that individuals imitate others when they expect social benefits from the other person (Wang & Hamilton, 2012). Based on this notion, it is reasonable to predict that group membership moderates automatic imitation especially when individuals feel affiliated with the in-group (Genschow & Schindler, 2016). Second, we investigated whether perceived similarity with the in- and the out-group moderates the relation between group membership and automatic imitation. Motor learning theories (e.g., Brass & Heyes, 2005; Greenwald, 1970; Heyes, 2010; Prinz, 1990, 1997) argue that automatic imitation is facilitated when perceived or actual similarity between actor and observer is increased. In line with this reasoning, we tested whether in-group members are imitated more strongly when they are perceived as more similar to oneself as compared with out-group members.

To test our predictions, we conducted six high-powered experiments. As all experiments had similar methods and tested the same hypothesis, we analyzed the data in a meta-analysis. In all experiments, automatic imitation was assessed with the imitation-inhibition task (Brass et al., 2000, 2001). In Experiments 1–4, we told US participants that they would see hands from US, German, or Chinese persons. To manipulate group membership in Experiments 5 and 6, we invited black and white participants and presented them with black and white hands. In all experiments, we assessed how similar participants perceive members of the in- and the out-group. Additionally, in Experiments 1, 2, 4, 5, and 6, we assessed how strongly participants felt affiliated with the in- and the out-group.

We report all experiments we ever conducted in this line of research, all manipulations, measures, and exclusions. All experiments were conducted in accordance with the ethical standards of the 1964 Declaration of Helsinki and in line with the ethical guidelines of the German Psychological Society (DGPs). The materials and data are available on the Open Science Framework (OSF; https://bit.ly/3sfyRvj). Experiment 6 was preregistered at Aspredicted (https://aspredicted.org/ug9zw.pdf ).

## Methods

### Participants

In total, 1538 participants took part in six experiments (see Table 1 for more details). Participants were recruited via Amazon’s Mechanical Turk (Experiments 1–5) or Prolific (Experiment 6). For each experiment, we aimed at detecting an effect size of at least dz = 0.25 for the difference in automatic imitation between in- and out-group members. To detect such an effect with a power of 1 − *β* = 0.85 and an Alpha probability of *α* = 0.05 (two-tailed) in a within-subject design, at least 146 participants are needed. With this power analysis in mind, we collected participants. In Experiments 5 and 6, we aimed at detecting even smaller effects with more power (i.e., effects of at least dz = 0.2 and power of at least 1 − *β* = 0.95). Consequently, we increased the sample sizes accordingly.

**Table 1:** Demographic information for Experiments 1–6

Exp.	Sample	*N* before exclusion	*N* exclusion Criterion 1	*N* exclusion Criterion 2	*N* exclusion Criterion 3	*N* after exclusion	% female	*M*_age_ (SD); range after exclusion
1	MTurk-USA	174	20	5	8	145	43.4	37.54 (11.98); 19–70
2	MTurk-USA	147	20	5	5	121	38.8	35.19 (11.25); 18–67
3	MTurk-USA	145	25	4	7	112	41.1	39.52 (11.22); 21–70
4	MTurk-USA	146	17	4	0	126	33.3	35.13 (9.37); 18–65
5	MTurk-USA	378	62	21	11	297	36.7	37.66 (11.25); 18–69
6	Prolific-UK	791	17	17	21	737	46.1	28.03 (9.49); 17–67

Note: Some participants met more than one of the exclusion criteria, so the total number of exclusion does not add up to the overall exclusion number. Criterion 1 = Participants for which less than 30% of trials remained after excluding erroneous trials, fast trials, and slow trials; Criterion 2 = Participants who reported to have used two hands instead of one during the imitation-inhibition task; Criterion 3 = participants who were non-US citizens (Experiments 1–4) or participants with a skin color that could neither be categorized as black or white (Experiments 5, 6)

We applied the following exclusion criteria across all experiments (see Table 1 for numbers of exclusions): We discarded participants (1) for which less than 30% of trials remained after excluding erroneous trials, fast trials (i.e., trials faster than 100 ms and trials more than 3 SDs below the participant’s mean), and slow trials (i.e., trials more than 3 SDs above the participant’s mean), (2) who reported to have used two hands instead of one during the imitation-inhibition task,^1^ and (3) who were non-US citizens (Experiments 1–4) or reported a skin color that could not be categorized as black or white (Experiments 5, 6).

### Procedure

We conducted all experiments online. The experiments were programmed in JavaScript using the jsPsych library (De Leeuw, 2015). In all experiments, we applied a similar procedure. First, all participants provided informed consent and were informed that participation was voluntary and that all answers were processed and stored anonymously. Next, they ran through the imitation-inhibition task (Brass et al., 2000, 2001). After the task, participants indicated perceived similarity of in- and out-group members (Experiments 1–6) as well as feelings of affiliation with the in- and out-group (Experiments 1, 2, 4, 5, and 6). Finally, they indicated basic demographics, were thanked, and dismissed.

In Experiments 3 and 4, we also assessed the inclusion of other in the self (IOS) scale (Aron et al., 1992) as well as pro-social attitudes towards in- and out-group members. As these scales were not central to our predictions, we report the associated results in the supplementary material only.

### Measures

Imitation-inhibition task: We applied a validated online version (Westfal et al., in preparation) of the imitation-inhibition task (Brass et al., 2000, 2001). The basic procedure of the online version is identical to previous research and produces similar strong and reliable results as when conducting the task in the laboratory. To accommodate the imitation-inhibition task to an online setting, it begins with a more detailed, stepwise practice procedure. First, participants ran through several different exercise blocks. In the first exercise block (ten trials), participants responded to randomly appearing number cues. That is, participants pressed and held down both the “g” key of their keyboard with their right index finger and the “h” key with their right middle finger. Afterwards, a fixation cross appeared for 500 ms, followed by the presentation of the number cue (i.e., either the number “1” or the number “2”). Participants had to lift their index finger in response to the number “1” and their middle finger in response to the number “2”. The number was presented until participants lifted a finger or for a maximum of 2000 ms. After each trial, participants received accuracy feedback. Participants had to repeat this first exercise block until they reached the threshold of at least eight correct trials.

In the second exercise block, we presented images of another person’s hand in addition to the numbers “1” and “2”. That is, when participants simultaneously pressed and held down the “g” and “h” key with their right index and middle finger, another person’s hand in mirrored and resting position appeared on the screen for 500 ms. We used the hands that were used in the original Brass et al. (2000, 2001) experiments (see stimuli on OSF; https://bit.ly/3sfyRvj). Afterwards, a picture of the same hand with either the lifted index or lifted middle finger was shown. Together with the lifted finger, either the number “1” or “2” was presented between the model’s index and middle finger for a maximum of 2000 ms or until participants lifted a finger. As in the first exercise block, participants had to respond by lifting the index finger in response to the number “1” and the middle finger in response to the number “2”. The setup of the trial structure resulted in two different trial types. On congruent trials, participants executed the same finger movement as the model and on incongruent trials, they executed another finger movement. After each trial, participants received accurate feedback. The exercise block consisted of 12 trials and was repeated until participants made less than four errors.

After successful completion of the two exercise blocks, the experimental blocks started. These blocks were similar to the second exercise block. However, we did not provide accurate feedback anymore. Moreover, we presented different hands to manipulate group membership. The way we manipulated group membership varied between experiments.

In Experiments 1–4 we told US citizens that they would see hands belonging to persons from different countries, including the USA, Germany, and China. To indicate which hand was from which country, the models were wearing colored gloves (i.e., blue, orange, and purple). We randomly varied across participants which color was matched with which country. To strengthen the manipulation, we presented the national flag of the respective country together with the hand (see Table 2 for screen shots of example trials). In Experiments 1–3, the flag was presented above the model’s hand. In Experiment 4, the flag was presented as a screen background. The size of the flags slightly varied between experiments (see Table 3 for details), because we wanted to test whether the salience of the flag influences the effect of group membership on automatic imitation. To strengthen the manipulation of the nationality, in Experiment 4, we presented in addition to the flag and the hand, a picture of a face of a US, German, or Asian-looking person. The pictures were taken from the Chicago face database (Ma et al., 2015). As US-looking face, we chose target face CFD-WM-213, as German-looking face target face CFD-WM-214, and as Asian-looking face target face CFD-AM-210. For all faces, we selected pictures with neutral facial expressions. A pretest confirmed that the faces were actually perceived as US, German, and Chinese, respectively (see supplementary material). The face pictures were presented at a size of 200 × 278 pixel. They appeared on the screen already 1500 ms before the hands were presented and remained on the screen until participants responded to the number cue.

**Table 2:**
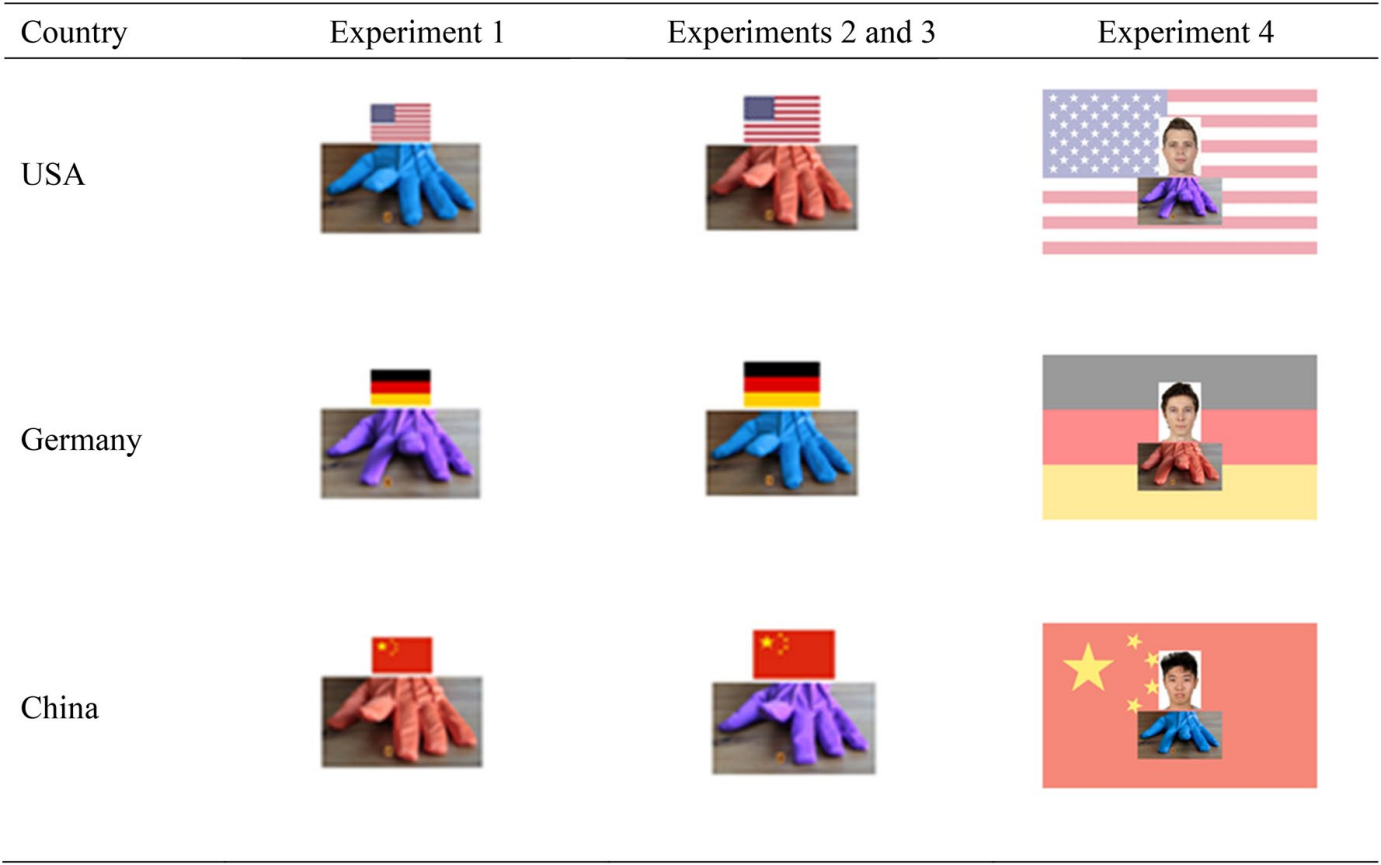
Screenshots of example trials used in Experiments 1–4

Note: Flag size slightly varied across Experiments 1–3 (for details, see Table 3)

**Table 3:** Specifications of stimuli and trials

Stimuli	Experiment 1	Experiment 2	Experiment 3	Experiment 4	Experiment 5	Experiment 6
	USA vs. Germany vs. China	USA vs. Germany vs. China	USA vs. Germany vs. China	USA vs. Germany vs. China	Black vs. white hand	Black vs. white hand
Number of trials per group	32 (16 congruent; 16 incongruent)	32 (16 congruent; 16 incongruent)	32 (16 congruent; 16 incongruent)	32 (16 congruent; 16 incongruent)	80 (40 congruent; 40 incongruent)	80 (40 congruent; 40 incongruent)
Number of blocks	3	3	3	3	4	4
Total number of trials	96	96	96	96	160	160
Erroneous trials	16.05%	14.83%	19.09%	12.19%	15.47%	7.47%
Trials faster than 100 ms	1.89%	2.44%	3.00%	1.76%	1.86%	0.11%
Trials faster than 3 SDs of participant’s mean	0.06%	0.09%	0.09%	0.08%	0.45%	0.02%
Trials slower than 3 SDs of participant’s mean	2.77%	2.55%	1.99%	1.21%	1.43%	1.08%
Flag size	150 × 90 px	200 × 120 px	200 × 120 px	1300 × 780 px	–	–-
Presentation time of base hand	500 ms	1250 ms	500 ms	500 ms	500 ms	500 ms

**Fig. 1:**
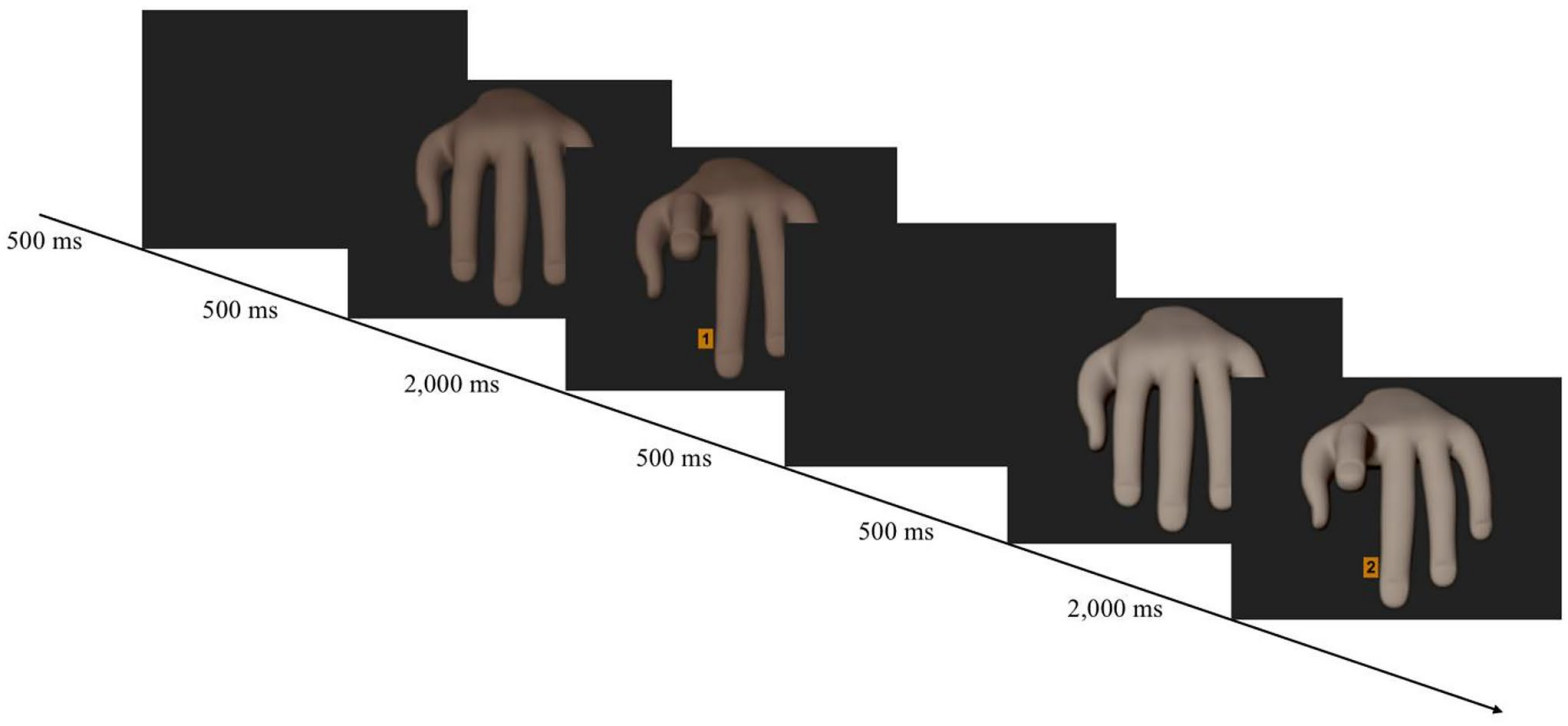
Trial structure of a congruent trial consisting of a black hand and an incongruent trial consisting of a white hand in Experiments 5 and 6

In principle, it could be that differences in nationality are not a relevant group dimension for participants. Thus, in Experiments 5 and 6, we changed the group membership manipulation by presenting black and white participants with black and white hands. We did not photograph actual hands, but created hands differing in color (black vs. white) using the open-source software Blender (Blender Foundation, 2020; Version 2.83.1) to manipulate 3D hands. The base hands were taken from Haupt (2012). With the help of the software GIMP (Version 2.10.10), we colored the hands accordingly. This approach allowed us to control for any potential confound such as the shape of the hand, its size, or the height of the finger lifting movement (see Fig. 1). To assess the group membership of the participants, we asked them at the end of the experiment to indicate their ethnic background.

To make sure that our results replicate when minor changes are made to the task, we varied a few aspects of the imitation-inhibition task between experiments (see Table 3 for details). First, we varied the presentation time of the base hand. Second, we slightly varied the number of blocks and the number of trials per block across experiments. In Experiments 1, 3, 4, 5, and 6, we presented each hand in each block in random order. In Experiment 2, we manipulated group membership block-wise. That is, we presented in each block a hand from one group. The order of the blocks varied randomly.

Perceived similarity: In all experiments, we measured perceived similarity between the self and members of the in- and out-group after the imitation-inhibition task. In Experiments 1–4, participants indicated on 7-point rating scales (1 = agree not at all; 7 = agree very much) their agreement with the following two statements: “An average American/German/Chinese person is similar to myself,” “An average American/German/Chinese person is different to myself.” To prepare data for analyses we averaged for each country the ratings to the first item with the reverse coded ratings to the second item, so that high values indicate high similarity. In Experiments 5 and 6, participants indicated their agreement on 7-point rating scales (1 = agree not at all; 7 = agree very much) with the following two statements: “An average white/black person is similar to myself”, “An average white/black person is different to myself”. To prepare data for analyses, for each group, we averaged the ratings to the first item with the reversed ratings to the second item, so that high values indicate high similarity.

Feelings of affiliation: In Experiments 1, 2, 4, 5, and 6 we assessed how affiliated participants felt with members of the in- and out-group by adopting the items used in previous research (e.g., Genschow & Schindler, 2016). In Experiments 1, 2, and 4, participants answered on 7-point rating scales (1 = not at all; 7 = very much) the following questions: “How strongly do you identify yourself with the USA/Germany/China?”, “How strongly do you share the same values as people from the USA/Germany/China?”. To prepare data for analyses, we averaged the ratings for each country, so that high values indicate a strong affiliation feeling. In Experiments 5 and 6, participants answered on 7-point rating scales (1 = not at all; 7 = very much) the following questions: “How strongly do you identify yourself with white/black people?” “How strongly do you share the same values as white/black people?” To prepare data for analyses, for each group, we averaged the answers to the first question with the answers to the second question, so that high values indicate a strong affiliation feeling.

## Results

To test our hypotheses, we conducted a fixed-effects meta-analysis across all experiments. We used a fixed-effects rather than a random-effects meta-analysis because the latter does not adequately control for false-positive rates when the number of included studies is small, as it is the case here (Borenstein et al., 2010; Field, 2001). This implies, however, that inferences are restricted to the set of included studies and do not necessarily generalize to other studies. That said, running exploratory random-effects meta-analyses instead of fixed-effects meta-analyses did not change any of the results for the present research. We analyzed the data with R (R Core Team, 2020; version 3.6.3) using the metafor package (Viechtbauer, 2010).

### Manipulation checks

In the first series of analyses, we conducted several manipulation checks.

Automatic imitation: First, we tested for the presence of automatic imitation by analyzing the latencies of the imitation-inhibition task (see supplementary material for error rate analyses). This analysis indicated that overall group conditions, participants responded faster to congruent than to incongruent trials, *d*_z_ = 1.81, SE = 0.04, 95% CI [1.73, 1.89], *z* = 43.22, *p* < 0.001.

Similarity: Second, we tested whether participants perceived members of the in-group as more similar to the self than members of the outgroup. When collapsing across all experiments (i.e., Experiments 1–6), the results indicate that participants perceived members of the in-group as more similar to themselves than members of the out-group, *d*_z_ = 0.70, SE = 0.03, 95% CI [0.65, 0.76], *z* = 24.53, *p* < 0.001. Results from Experiments 1 to 4 show that US MTurkers perceived US citizens as more similar to themselves than German citizens, *d*_z_ = 0.74, SE = 0.05, 95% CI [0.64, 0.84], *z* = 14.66, *p* < 0.001, or Chinese citizens, *d*_z_ = 1.03, SE = 0.06, 95% CI [0.92, 1.14], *z* = 18.62, *p* < 0.001.

Feelings of affiliation: Third, we tested whether participants indicated stronger feelings of affiliation for in-group members than for out-group members. When collapsing across all experiments (i.e., Experiments 1–6), the results indicate that participants reported stronger feelings of affiliation with members of the in-group than with members of the out-group *d*_*z*_ = 1.00, SE = 0.03, 95% CI [0.94, 1.07], *z* = 30.39, *p* < 0.001. Likewise, when analyzing only the results from Experiments 1, 2, 3, and 4, US MTurkers reported stronger feelings of affiliation with US citizens than with German citizens, *d*_z_ = 1.18, SE = 0.07, 95% CI [1.05, 1.30], *z* = 17.82, *p* < 0.001, or Chinese citizens, *d*_z_ = 1.43, SE = 0.07, 95% CI [1.28, 1.57], *z* = 19.77, *p* < 0.001.

### Main analyses

**Fig. 2:**
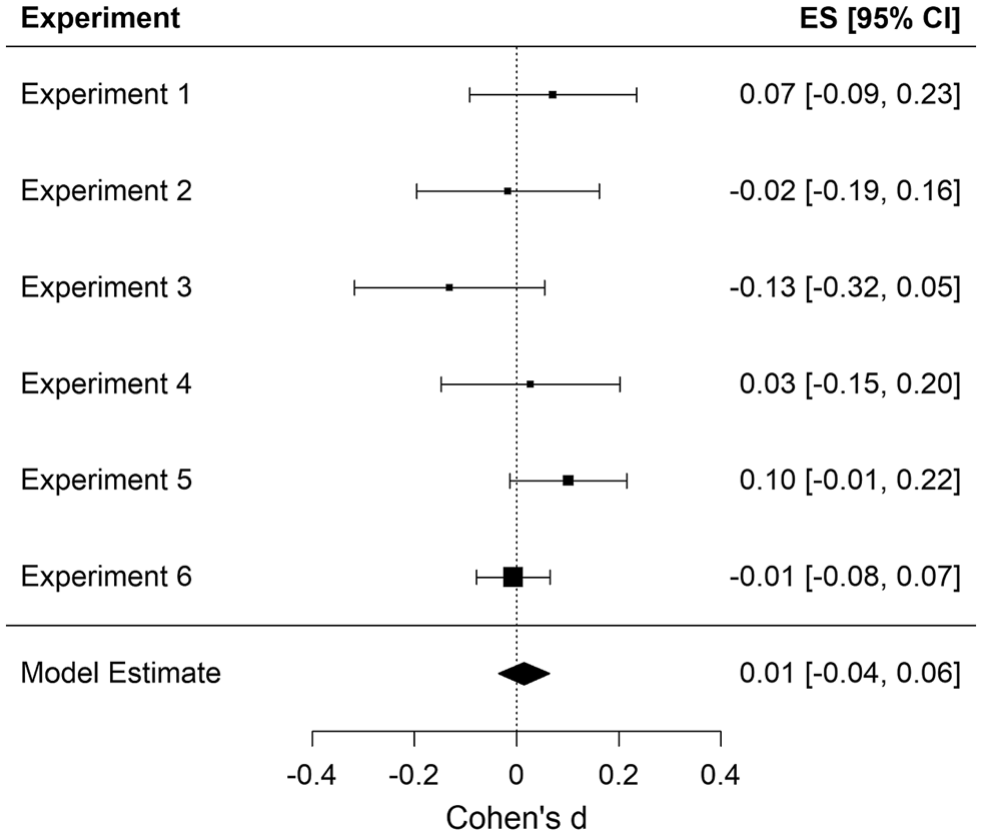
Forest plot for the difference in automatic imitation between in- and out-group members

Automatic imitation of in- and out-group members: To test whether participants imitated members of the in-group more strongly than members of the out-group, we compared participants’ congruency effect (i.e., the difference between congruent and incongruent trials) for in- and out-group trials (for more details, see Table 4). We restricted our analyses to latencies, as this measure is more reliable than the error rates (Genschow et al., 2017). Nevertheless, we report the same analyses for the error rates in the supplementary material. The results across Experiments 1–6 indicate that the congruency effect for in-group trials did not differ from the congruency effect for out-group trials, *d*_z_ = 0.02, SE = 0.03, 95% CI [− 0.04, 0.07], *z* = 0.57, *p* = 0.567. As can be seen in Fig. 2, the group membership effect was not significant in any of the experiments.

**Table 4:** Mean values and standard deviations of congruent and incongruent trials within in- and out-group trials

Exp.	Reaction times [ms]				Error rates [%]			
	In-group		Out-group		In-group		Out-group	
	Mean congruent trials (SD)	Mean in-congruent trials (SD)	Mean congruent trials (SD)	Mean in-congruent trials (SD)	Mean congruent trials (SD)	Mean in-congruent trials (SD)	Mean congruent trials (SD)	Mean in-congruent trials (SD)
1	564.09 (189.14)	624.68 (203.13)	567.60 (188.31)	622.61 (193.97)	2.15 (3.78)	7.07 (6.32)	1.98 (4.03)	6.10 (4.84)
2	625.42 (224.44)	702.78 (232.31)	637.42 (225.64)	715.86 (236.62)	1.06 (2.51)	5.13 (5.36)	1.04 (2.07)	5.14 (4.25)
3	629.94 (219.44)	685.28 (230.96)	629.21 (218.99)	693.62 (230.45)	3.03 (5.43)	6.75 (7.03)	2.72 (3.72)	6.34 (5.31)
4	686.05 (275.46)	759.47 (287.99)	679.79 (263.85)	751.38 (269.13)	1.30 (3.76)	4.53 (5.34)	1.19 (2.47)	4.09 (4.07)
5	761.49 (285.91)	839.02 (290.89)	766.38 (288.36)	838.64 (291.13)	1.72 (4.27)	5.76 (6.52)	1.59 (3.08)	6.03 (6.43)
6	473.73 (92.89)	550.51 (102.39)	475.38 (97.42)	552.40 (105.15)	1.01 (1.88)	5.19 (4.96)	1.06 (1.83)	5.36 (5.05)

Furthermore, the results across Experiments 1–4 indicate that the US MTurkers’ congruency effects for US hands (i.e., the in-group) did not differ from the congruency effects for German, *d*_z_ = 0.01, SE = 0.05, 95% CI [− 0.08, 0.10], *z* = 0.16, *p* = 0.873, or Chinese hands, *d*_z_ = − 0.01, SE = 0.05, 95% CI [− 0.10, 0.07], *z* = − 0.32, *p* = 0.751.

Moderator analyses: In a final series of analyses, we tested whether perceived similarity and feelings of affiliation moderate the influence of group membership on automatic imitation. To prepare data for analyses, we first computed across all experiments an in–out group imitation effect by subtracting the congruency effect for out-group members from the congruency effect of in-group members. Second, we computed the difference between perceived similarity for the in-group and the out-group as well as the difference between feelings of affiliation with the in- and out-group. Afterwards, we ran meta-analytical correlational analyses across all experiments. The results indicate that neither the in–out group similarity score (*r* = 0.01, *p* = 0.672), nor the affiliation score (*r* = -0.01, *p* = 0.807) correlated with the in–out group imitation score.

## Discussion

A prominent prediction derived from different theories of imitation is that in-group members are imitated more strongly than out-group members. However, past research investigating this prediction produced rather mixed results. While some researchers found support for this predictions (Genschow & Schindler, 2016; Gleibs et al., 2016), others found the opposite (Rauchbauer et al., 2015, 2016) and yet others found no difference between the imitation of in- and out-group members (De Souter et al., 2021). To shed further light onto the group membership prediction, we tested it in six high-powered experiments (total *N* = 1538). Across all experiments, the results indicated that group membership does not influence automatic imitation.

### Reasons for the null finding

These results raise the question of why group membership does not modulate automatic imitation. First, one could argue that in our experiments group membership was not salient enough. However, this is rather unlikely, as we took great care in making clear to which group each presented hand belonged either by presenting the respective national flag together with the hand or by coloring the hand white or black. As the stimuli blatantly varied in terms of their group membership, we do not regard it as plausible that group membership was not salient enough during the imitation task. Moreover, as we found large differences between the in- and out-groups in terms of rated similarity and feelings of affiliation, it is also apparent that the groups represented meaningful and important social categories.

Second, it might be that hidden moderators influence the relationship between group membership and automatic imitation. We tested two of the most prominent moderators (i.e., similarity and feelings of affiliation), but could not find support for their influence. This is in line with related research from De Souter and colleagues (2021) who did not find an influence of affiliation motives on the relation between group membership and imitation either. In addition, De Souter et al. tested whether differences in directed attention to the in- versus out-group may moderate the relation between group membership and automatic imitation, but did not find support for this hypothesis. Nevertheless, other factors could still moderate the relationship between group membership and automatic imitation. For instance, some researchers suggested that perceiving anger in the other person (Rauchbauer et al., 2016) or being in a cooperation versus competition mindset (Gleibs et al., 2016) may influence the impact of group membership on automatic imitation. Our data do not allow testing this possibility, as they merely show that group membership itself does not influence automatic imitation. Thus, future research may investigate further moderators to test whether the relation between group membership and automatic imitation can be detected within specific conditions only.

Third, it is possible that group membership does not affect automatic imitation at all. Indeed, we regard this explanation as plausible, since we tested the prediction in large samples by manipulating group membership in two of the most explicit and extreme ways (i.e., nationality and ethnic group affiliation). If group membership does not play a role in such a setting, it most likely does not modulate automatic imitation.

### Theoretical implications

The finding that group membership does not influence automatic imitation has important implications for theories explaining social modulation of automatic imitation. Motivational theories of imitation (Chartrand & Dalton, 2009; Wang & Hamilton, 2012) argue that individuals use imitation as a tool to affiliate with others. As individuals have the general tendency to affiliate more with the in-group than with the out-group (Van Der Schalk et al., 2011), in-group members should be more strongly imitated than out-group members. Based on this reasoning, the relationship between group membership and automatic imitation should be moderated by feelings of affiliation. Motor learning theories (e.g., Brass & Heyes, 2005; Greenwald, 1970; Heyes, 2010; Prinz, 1990, 1997) predict that in-group members should be imitated more strongly than out-group members because in-group members are perceived as more similar to the self than out-group members. Thus, differences in perceived similarity should moderate the influence of group membership on automatic imitation. Our results do neither support the assumptions derived from motivational theories nor the ones derived from motor learning theories because (1) group membership did not influence automatic imitation and (2) neither feelings of affiliation nor perceived similarity moderated the relationship between group membership and automatic imitation. It is important to note that these results do not question the general validity of motivational and motor learning theories of imitation, but rather limit the range of their predictions by suggesting that the postulated principles of social modulation do not translate to the influence of group membership.

Interestingly, the conclusion that automatic imitation is not affected by group membership fits to several other recent findings illustrating the resilience of automatic imitation against social modulations. For example, recent research found difficulties in replicating correlations between automatic imitation and different interindividual differences including autism-like traits, narcissism, empathy, and perspective taking (Butler et al., 2015; Cracco, Bardi, et al., 2018; Galang & Obhi, 2020; Genschow et al., 2017; Müller et al., 2013; Newey et al., 2019). Likewise, Khemka et al. (2020) could not replicate the finding that sitting in front of a mirror reduces automatic imitation (Spengler et al., 2010).

Together with this literature, our findings contribute to a current debate in the literature about the degree to which automatic imitation, and the imitation-inhibition task, in particular, is driven by social processes. Ramsey (2018) argues that automatic imitation in the imitation-inhibition task is the result of a combination of several different underlying processes, which are neither necessarily related to imitation, nor to other forms of social behavior. In contrast to this view, Cracco and Brass (2019) argue that the imitation-inhibition task measures covert imitative response tendencies associated with some (but not all) types of overt imitation. Based on this view, one could conclude that imitation is a social process. However, this does not necessarily mean that automatic imitation can be socially modulated. Our results suggest that at least in the case of group membership, automatic imitation is not socially modulated. At the same time, it is important to note that other experiments indicate that the imitation-inhibition task can be socially modulated. For example, research demonstrated that individuals engage in stronger imitative behavior when they observe human as compared to non-human actions (Klapper et al., 2014; Liepelt & Brass, 2010; Press et al., 2005, 2006), when they observe social as compared to antisocial gestures (Cracco, Genschow, et al., 2018), or when they focus on others as compared to the self (Cracco et al., 2019; Genschow, Schuler, et al., 2019; Hogeveen & Obhi, 2011; Leighton et al., 2010; Wang & Hamilton, 2013). As some (but not all) of these studies were based on a small number of participants, future research should aim at replicating these findings with larger samples. The results will further our understanding on the social processes underlying automatic imitation.

### Limitations and future directions

Besides these implications, several limitations of our experiments need to be discussed. First, one may argue that the influence of group membership on automatic imitation is smaller than expected and that we did not have sufficient power to detect such a small effect. In this respect, it is important to note that each of our experiments (total *N* = 1538) was powered to detect even a small effect of dz = 0.25. Moreover, Experiments 5 and 6 included a sufficient number of participants to detect even effects that are smaller than dz = 0.2 with more than 90% of power.

Second, in contrast to previous research, we did not use artificial groups or minimal group paradigms to manipulate group membership but instead assessed existing groups such as persons from different countries or persons differing in skin color. Nevertheless, it could be that our manipulations were still too artificial to detect the predicted effect. In Experiments 1–4, participants needed to associate the hands in the gloves with the persons from different nationalities. In Experiments 5 and 6, we circumvented this issue by presenting participants with white and black hands. Yet, to control for any potential confound, we created the stimuli with a computer software, which might have made the hands look artificial. Thus, in future research, one may use photographs of actual hands instead.

Third, as we tested the influence of group membership in online settings in which participants did not personally interact with the imitated person, it could be that participants’ motivation to affiliate was reduced. However, it is important to note that we found significant differences between in- and out-group members in terms of perceived similarity and feelings of affiliation despite the online character of our experiments. Moreover, in lab experiments that use the same imitation-inhibition task, participants do not personally interact with the other person either. Thus, we regard it as rather unlikely that the found null effects can be explained by the online setting of our experiments. Nevertheless, it might well be that the lack of personal interaction is the reason why automatic imitation is not modulated by group membership. Thus, future research could assess the effect of group membership by using other imitation tasks, such as mimicry tasks (Chartrand & Bargh, 1999; Genschow et al., 2018), in which participants personally interact with a confederate.

### Summary

A key prediction derived from different theories of automatic imitation suggests that imitation is stronger when observing actions from in-group members, as compared with out-group members. While previous research testing this prediction produced mixed results, our results clearly demonstrate that group membership per se does not influence automatic imitation. Moreover, our results show that neither perceived similarity nor feelings of affiliation moderate the influence of group membership on automatic imitation. These results challenge to some degree some of the predictions derived from motivational and learning theories of imitation.

## Supplementary Information

Below is the link to the electronic supplementary material.

Supplementary file1 (DOCX 27 kb)

## Footnotes

1 Including only participants who used the right hand does not change the pattern of results.
