# Supplementary material for: Group membership does not modulate automatic imitation
Source: Psychol Res. 2021 Jun 9;86(3):780–91. doi: 10.1007/s00426-021-01526-1 (PMC8942900; doi:10.1007/s00426-021-01526-1)
Supplement: Supplementary file 1 — Supplementary file1 (DOCX 27 kb) [file 426_2021_1526_MOESM1_ESM.docx]

Supplementary material for

Group membership does not modulate automatic imitation

Oliver Genschow (University of Cologne)

Mareike Westfal (University of Cologne)

Emiel Cracco (Ghent University)

Jan Crusius (University of Cologne)

**This PDF file includes**

Further methodological specifications

Additional analyses

References

**Experiment 3 and 4**

**Facial Stimuli**

In Experiment 4, we presented in addition to the hands, face pictures of either US, German, or Asian looking persons. We let ten participants (7 women, 3 men), with an age between 20 and 60 (*M* = 31.60, *SD* = 15.11), rate the faces. Participants rated for each person the degree to which it looked typically American, German, and Chinese on 7-point rating scales (1 = *not at all*; 7 = *very much*). Table 1 illustrates the average ratings for each face.

**Table 1**

A*verage Ratings of the Three Faces Used in Experiment 4*

| Target Face | American [*M, SD*] | German [*M, SD*] | Chinese [*M, SD*] |
| --- | --- | --- | --- |
| WM-213 | 5.50, 1.72 | 3.70, 1.70 | 1.00, 0.00 |
| WM-214 | 4.30, 1.90 | 5.60, 1.35 | 1.10, 0.32 |
| AM-210 | 1.40, 0.70 | 2.10, 1.20 | 6.30, 0.82 |

*Note*. For all faces, from the Chicago Face Database, we took the face with a neutral expression.

**Additional Measures**

In Experiments 3 and 4, we assessed two further measures besides feelings of affiliation and perceived similarity. First, we assessed the Inclusion of Other in the Self (IOS) scale (Aron et al., 1992). Participants saw for each country a Venn diagram-style measure that consisted of seven pairs of increasingly overlapping circles in which one circle represented the self and the other circle represents the average person from another country (i.e. US, Germany, and China). Participants indicated for each country the degree to which their self overlapped with the other person. To prepare the data for analysis, we computed an IOS index. First, we averaged the ratings for both out-groups (i.e. Germany and China) and then subtracted the averaged out-group rating from the in-group rating. High values indicate relatively more overlap of the self with members from the in-group as compared to the out-group.

Second, we assessed pro-social attitudes towards members of the in-group and the out-group. In particular, participants answered on 7-point ratings scales (0 = *0 Dollars*; 6 = *6 Dollars*) the following question: “Imagine you would get 6 Dollars. How much would you donate to a US/ German/ Chinese charity?”. To prepare the data for analysis, we computed a pro-social attitude index by averaging the ratings for the two out-groups (i.e., Germany and China). Afterwards, we subtracted this average from the rating of the in-group (i.e. USA). High values indicate a stronger prosocial attitude towards the in-group as compared with the out-group.

**Results**

First, we computed an in-out group imitation index by subtracting the congruency effect for out-group members from the congruency effect for in-group members. Afterwards, we correlated this imitation index with the IOS index and the pro-social attitude index. The imitation index did not correlate significantly with the IOS and pro-social attitude indices in any of the experiments (see Table 2 for an overview), except for the reaction times IOS index in Experiment 4, which, however, was hardly significant and opposite to the expected direction.

**Table 2**

*Intercorrelations (r) Between the Imitation Indices and the IOS Index As Well As the Pro-Social Attitude Index in Experiments 3 and 4*

| Imitation index | IOS | Pro-social attitude |
| --- | --- | --- |
| RT in Experiment 3 | .10 | .06 |
| ER in Experiment 3 | -.11 | -.09 |
| RT in Experiment 4 | -.16^*^ | -.01 |
| ER in Experiment 4 | .03 | -.01 |

*Note.* RT (Reaction times); ER (Error rates)

* *p* < .05; ** *p* < .01; *** *p* < .001

**Error Rate Analyses**

In additional meta-analyses, we tested the prediction that group membership moderates automatic imitation by analyzing the error rates of the imitation-inhibition task. First, we tested for the presence of automatic imitation. This analysis indicated that over all group conditions, participants in Experiments 1 to 6 made fewer errors on congruent than on incongruent trials, *d*_z_ = 1.00, SE = 0.03, 95% CI = [0.94, 1.06], *z* = 32.03, *p* < .001

Second, we tested whether the congruency effect of the error rates differed between in- and out-group trials. The meta-analytical analyses indicate that the mean values did not differ from each other, *d_z_* = -0.01, SE = 0.03, 95% CI = [-0.06, 0.04], *z* = -0.31, *p* = .758.

Finally, we investigated whether perceived similarity and feelings of affiliation moderate the influence of group membership on automatic imitation measured with the error rates. To prepare data for analyses, we computed across all experiments an in-out group imitation effect by subtracting the congruency effect for out-group members from the congruency effect of in-group members. Afterwards, we computed the difference between perceived similarity for the in-group and the out-group as well as the difference between the feeling of affiliation with the in- and out-group. Meta-analytical correlational analyses across all experiments indicate that the in-out group similarity score did not significantly correlate with the in-out group imitation score (*r* = -.03, *p* = .252). However, we the affiliation score correlated significantly with the in-out group imitation score (*r* = -.07, *p* = .008). However, the correlation went into the opposite direction as we had predicted.

**References**

Aron, A., Aron, E. N., & Smollan, D. (1992). Inclusion of other in the self scale and the structure of interpersonal closeness. *Journal of Personality and Social Psychology, 63*, 596-612.
